# The association of midregional pro-adrenomedullin (MR-proADM) at ICU admission and fluid overload in patients post elective cardiac surgery

**DOI:** 10.1038/s41598-024-71918-x

**Published:** 2024-09-08

**Authors:** Carmen A. Pfortmueller, Isabelle Ott, Martin Müller, Darius Wilson, Joerg C. Schefold, Anna S. Messmer

**Affiliations:** 1 Department of Intensive Care Medicine, Inselspital, Bern University Hospital, University of Bern, Bern, Switzerland; 2 Department of Emergency Medicine, Inselspital, Bern University Hospital, University of Bern, Bern, Switzerland; 3 Shock, Organ Dysfunction and Resuscitation Research Group, Vall d’Hebron Institute of Research, Barcelona, Spain

**Keywords:** MR-proADM, Cardiac surgery, Fluid overload, Critical care, Cardiovascular diseases, Predictive markers, Prognostic markers

## Abstract

Postoperative fluid overload (FO) after cardiac surgery is common and affects recovery. Predicting FO could help optimize fluid management. This post-hoc analysis of the HERACLES randomized controlled trial evaluated the predictive value of MR-proADM for FO post-cardiac surgery. MR-proADM levels were measured at four different timepoints in 33 patients undergoing elective cardiac surgery. Patients were divided into FO (> 5% weight gain) and no-FO at ICU discharge. The primary outcome was the predictive power of MR-proADM at ICU admission for FO at discharge. Secondary outcomes included the predictive value of MR-proADM for FO on day 6 post-surgery and changes over time. The association between MR-proADM and FO at ICU discharge or day 6 post-surgery was not significant (crude odds ratio (cOR): 4.3 (95% CI 0.5–40.9, *p* = 0.201) and cOR 1.1 (95% CI 0.04–28.3, *p* = 0.954)). MR-proADM levels over time did not differ significantly between patients with and without FO at ICU discharge (*p* = 0.803). MR-proADM at ICU admission was not associated with fluid overload at ICU discharge in patients undergoing elective cardiac surgery. MR-proADM levels over time were not significantly different between groups, although elevated levels were observed in patients with FO.

## Introduction

Postoperative fluid overload in patients undergoing cardiac surgery is common^1–3^. During and after cardiac surgery, intravenous fluids are frequently administered to optimize oxygen delivery and perfusion pressures^4,5^. In addition, patients undergoing cardiac surgery on cardiopulmonary bypass are prone to inflammation and development of systemic inflammatory response syndrome (SIRS) and thus susceptible for capillary leakage^6–9^. This further perpetuates fluid accumulation in these patients. Importantly, fluid overload after surgery has negative effects on outcomes of respective patients^10,11^. However, appropriate fluid therapy and hemodynamic management is challenging in cardiac surgery and often complicated by a multitude of factors on the patient, surgical and technical sides that strongly influence hemodynamic parameters^4,12^. Hence, predicting fluid sequestration could be clinically relevant in this patient population in an effort to guide fluid administration post cardiac surgery.

Several serum and urinary biomarkers, such as stress hormones (cortisol or catecholamines), hormones regulating the water retention (renin-angiotensin II-aldosterone system), pro-arginine vasopressin (also known as copeptin), atrial natriuretic peptides, and endothelium-derived factors (mid-regional proadrenomeddulin; MR-proADM) have been studied to assess respective predictive values of fluid overload in critically ill patients, as well partly in patients post cardiac surgery^13–15^.

MR-proADM, the stable surrogate marker of adrenomedullin (ADM), is suggested to play a major role in reducing vascular permeability and is released as a response to hyper-permeability in the microcirculation and subsequent capillary leakage^16–18^. It acts as counter-regulator for deranged microcirculation, tissue ischemia, and tissue damages by inflammatory factors^19^. Furthermore, MR-proADM was shown a good predictor of fluid overload in intensive care, including patients with septic shock, severe brain trauma, non-cerebral trauma, and aneurysmal subarachnoid haemorrhage^13,14^. In patients undergoing cardiac surgery, biomarkers such as ADM are considered of particular interest, as these patients are exposed to perioperative oxidative stress secondary to major surgical trauma in combination with cardiopulmonary bypass^20,21^. In patients with heart failure and with acute ischemic heart disease, pro-ADM was shown to have diagnostic and prognostic value, suggesting that it may be comparable to brain natriuretic peptide (BNP) a marker of fluid overload^22–26^. Therefore, the aim of this study was to explore the predictive value of MR-proADM for fluid overload after elective cardiac surgery.

## Method

### Study design and setting

This study was a post-hoc analysis of the HERACLES trial, a monocentric, prospective, randomised, double-blind clinical trial, assessed the impact of a single low volume infusion of hypertonic saline on total fluids administered in low-risk cardiac surgical patients^3^. The study was conducted from 28th of February 28, 2018 until the 27th of August, 2019 at the Department of Intensive Care Medicine and Cardiovascular Surgery at the University Hospital Bern, Inselspital. The study was approved by the local Ethics Committee of the Canton Bern (Project No 2016-01,039), and was carried out in accordance with the Declaration of Helsinki and the International Conference of Harmonisation (ICH) guidelines for Good Clinical Practice (GCP). Written consent was achieved from all participants prior to inclusion.

The study design and study populations were reported elsewhere^3,27,28^. In brief, all patients scheduled for elective coronary and/or valvular cardiac surgery were screened for eligibility. Patients were excluded when any of the following criteria were meet: severe heart failure (LVEF < 30%), severe renal dysfunction (estimated glomerular filtration rate < 30 mL/min/m^2^), systemic steroid therapy at baseline, chronic liver disease, an active infection or sepsis, and/or pregnancy or breastfeeding.

Patients included in the HERACLES trial were undergoing a second postoperative safety screening prior to randomisation. Patients with elevated sodium levels or chloride levels, as well as patients requiring mechanical circulatory support (extra-corporeal membrane oxygenation (ECMO), or impella device) postoperatively, were not randomized to receive the study intervention and were treated at the discretion of the attending physician (standard of care). All other patients were randomised to receive either 5 mL/kg body weight of 7.3% NaCl or the same amount of 0.9% NaCl over 60 min. Study groups in this study thus consist in (A) hypertonic saline (B) 0.9% saline and (C) standard of care groups (excluded after secondary post-operative safety screening, see above). The present substudy comprised 33 consecutive enrolled patients. In this group we additionally collected plasma samples to evaluate the predictive value of MR-proADM at ICU admission on fluid overload at ICU discharge^3^.

### Blood sampling and MR-proADM measurement

In all patients, plasma samples were drawn at D-1 (day prior to surgery), D0 (admission to ICU), and D1 (day 1 postoperative or ICU discharge), and D6 days after surgery. All blood samples were centrifuged at 23° for 7 min, 3000 g and frozen within 30 min at − 80 °C at the central liquid biobank at the University Hospital Bern, Switzerland.

As measurement of ADM is unreliable due to a rapid degradation by proteases and thus short half-life, the surrogate marker MR-proADM, a fragment of 48 amino acids were analysed for this study^29^. The MR-proADM levels represent proportionally the levels and activity of Adrenomedullin^29^. MR-proADM concentrations were measured in an automated Kryptor analyser using Time-Resolved Amplified Cryptate Emission (TRACE) technology (Kryptor; Thermo Fisher Scientific (BRAHMS GmbH), Hennigsdorf, Germany). According to the manufacturer, the lower detection limit was 0.05 nmol/L, while the limit of quantitation (LOQ) was 0.23 nmol/L.

### Outcome

Primary outcome of this sub analysis was the predictive value of MR-proADM value at ICU admission on fluid overload at ICU discharge. Secondary outcomes were predictive value of MR-proADM on fluid overload at day 6 after surgery. MR-proADM values over time (in FO and non FO group). Fluid overload was defined as ≥ 5% of weight gain and calculated as (total input − total output/ weight) *100^30^.

### Statistical analysis

The statistical analysis was performed with STATA 18.1 (StataCorp, The College Station, Texas, USA).

Depending on normality testing (Shapiro Wilk) median (IQR) respectively mean (SD) are shown for continuous variables. *P*-values were obtained by Wilcoxon rank sum test respectively unpaired T-test for comparison between two groups. Categorical variables are shown with number (%) in each category, *p*-values obtained by Chi-squared test. Fisher’s exact test was additionally calculated, when the expected frequency of one cell was less than 5. For graphical representation, the distribution of MR-proADM was shown in different groups using violin plots^31^, respectively the mean with SD over time. Univariable logistic regression was used to study the association between MR-proADM value at ICU admission and fluid overload (i) at ICU discharge and (ii) at day 6 post surgery. In addition, a sensitivity analysis was performed to assess the association of MR-proADM at ICU admission and fluid balance at ICU discharge as well as weight gain on day 6 post-surgery, both as continuous outcomes. Linear and non-linear relationships were assessed to study the associations using linear regression analysis and restricted cubic spline regression. A repeated measures mixed model to analyse MR-proADM values over time in the two fluid overload groups. Univariable linear regression analysis was used to evaluate the association of MR-proADM with (i) norepinephrine use (dose and duration) and (ii) duration of mechanical ventilation. A *p*-value was set to 0.05 for significance. Missing values were reported, no imputation was performed as the dataset was almost complete.

## Results

The subgroup of 33 patients had comparable baseline characteristics compared to the total HERACLES population (N = 165), and thus constitutes a representative sample (Supplemental Table S1). The median age of the study population was 67 years (Interquartile Range (IQR) 59–72) and 26 (78.8%) were male. Table 1 shows baseline characteristics stratified by fluid overload on ICU discharge. The median length of ICU stay was 0.88 days (IQR 0.84–0.91). Twenty-two (66.7%) patients fulfilled the definition of fluid overload at ICU discharge. Table 2 shows all outcomes. Results from regression models can be found in the Supplemental file (Tables S2–S9).

**Table 1:** Baseline characteristics.

Fluid overload				
Characteristics	Overall (N = 33^1^)	No (N = 11^1^)	Yes (N = 22^1^)	*p*-value
Demographics				
Sex				0.228
Male	26 (79%)	10 (91%)	16 (73%)	
Female	7 (21%)	1 (9%)	6 (27%)	
Age (years)	66.3 (9.0)	69.4 (7.5)	64.8 (9.4)	0.174
Height	172.5 (10.1)	174.3 (6.2)	171.6 (11.6)	0.482
Weight	85.9 (74.1, 98.8)	87.9 (80.9, 91.5)	85.7 (72.6, 104.7)	0.962
BMI	27.9 (4.5)	28.2 (3.4)	27.8 (5.0)	0.823
Randomisation group				
Hypertonic saline	14 (42%)	3 (27%)	11 (50%)	0.3
Normale saline	11 (33%)	3 (27%)	8 (36%)	0.7
Standard of care	8 (24%)	5 (45%)	3 (14%)	0.08
ASA classification				
4	33 (100%)	11 (100%)	22 (100%)	
Comorbidities				
LVEF (%) at baseline	60 (50, 65)	60 (55, 66)	60 (50, 65)	0.367
Known chronic renal disease	2 (6.1%)	0 (0%)	2 (9.1%)	0.302
Surgery/Anaesthesia characteristics				
Duration anaesthesia (h)	5.7 (1.2)	5.4 (0.9)	5.8 (1.4)	0.414
Duration surgery (h)	3.9 (1.1)	3.8 (0.9)	3.9 (1.2)	0.685
Duration ECC	1.40 (1.28, 2.37)	1.31 (1.25, 1.70)	1.49 (1.29, 2.41)	0.3

^1^Mean (SD); n (%); Median (IQR); BMI, body mass index; LVEF, left ventricular ejection fraction; ASA, American Society of Anaesthesiologists; LOS, length of stay; ECC, extra corporeal circulation; FO, fluid overload; MV, mechanical ventilation; ICU, intensive care unit.

**Table 2:** Outcomes.

Fluid overload				
Characteristics	Overall (N = 33^1^)	No (N = 11^1^)	Yes (N = 22^1^)	*p*-value
Post-surgery characteristics				
Cumulative fluid balance at ICU discharge	1964.0 (1513.5)	1433.2 (1631.6)	2229.4 (1414.8)	0.157
Cumulative fluids perioperative	8.078 (6.002, 9.281)	6.337 (5.374, 9.175)	8.187 (7.833, 9.244)	0.3
FO post-surgery	7 (23%)	2 (20%)	5 (24%)	> 0.9
FO at day 6	3 (9.1%)	0 (0%)	3 (14%)	0.5
Weight at day 6	83.5 (16.0)	84.3 (12.1)	83.0 (18.0)	0.833
Duration of MV (d)	0.6 (0.5, 0.6)	0.5 (0.4, 0.6)	0.6 (0.5, 0.8)	0.647
Duration of vasopressors (h)	0.3 (0.2, 0.7)	0.2 (0.2, 0.2)	0.4 (0.3, 0.7)	0.012
Total vasopressor dose (mg)	0.3 (0.1, 2.0)	0.1 (0.1, 0.2)	0.4 (0.1, 2.5)	0.089
MR-proADM Values (nmol/L)				
MR-proADM 1 day preoperative (n = 33)	0.6 (0.5, 0.7)	0.6 (0.5, 0.6)	0.6 (0.5, 0.7)	0.954
MR-proADM at ICU admission (n = 33)	0.9 (0.7, 1.3)	0.9 (0.6, 1.2)	1.1 (0.9, 1.4)	0.194
MR-proADM day 1 postoperative (n = 33)	1.4 (0.5)	1.3 (0.4)	1.5 (0.6)	0.350
MR-proADM day 6 postoperative (n = 29)	0.9 (0.7, 1.1)	0.9 (0.7, 1.1)	0.9 (0.7, 1.4)	0.614
Outcomes				
LOS ICU	0.88 (0.84, 0.91)	0.88 (0.84, 0.89)	0.88 (0.83, 0.91)	0.730
LOS hospital	8.0 (7.4, 11.0)	8.1 (7.0, 12.0)	8.0 (7.4, 10.1)	0.660

^1^Mean (SD); n (%); Median (IQR); BMI, body mass index; LVEF, left ventricular ejection fraction; ASA, American Society of Anaesthesiologists; DHCA, deep hypothermic circulatory arrest; ECC, extra corporeal circulation.

### MR-proADM and fluid overload

**Fig. 1:**
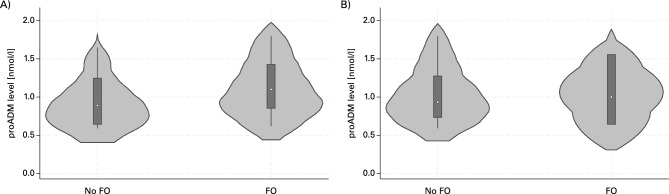
Violin chart MR-proADM levels at ICU admission in patients with and without fluid overload. (**A**) Fluid overload assessed at ICU discharge. (**B**) Fluid overload assessed at day 6 post surgery. FO = Fluid overload.

There was no significant association between MR-proADM at ICU admission and fluid overload at ICU discharge (crude odds ratio (cOR) 4.3 (95% CI 0.5–40.9, *p* = 0.2), and no association with the development of fluid overload at day 6 postoperative (cOR 1.1 (95% CI 0.04–28.3, *p* = 0.954). No significant differences were observed, when adjusted to randomisation group (hypertonic vs normal saline vs standard of care). The violin plot in Fig. 1 depicts the distribution of MR-proADM values at ICU admission to predict fluid overload at ICU discharge (A) and on day 6 post-operative (B).

### Sensitivity analysis

**Fig. 2:**
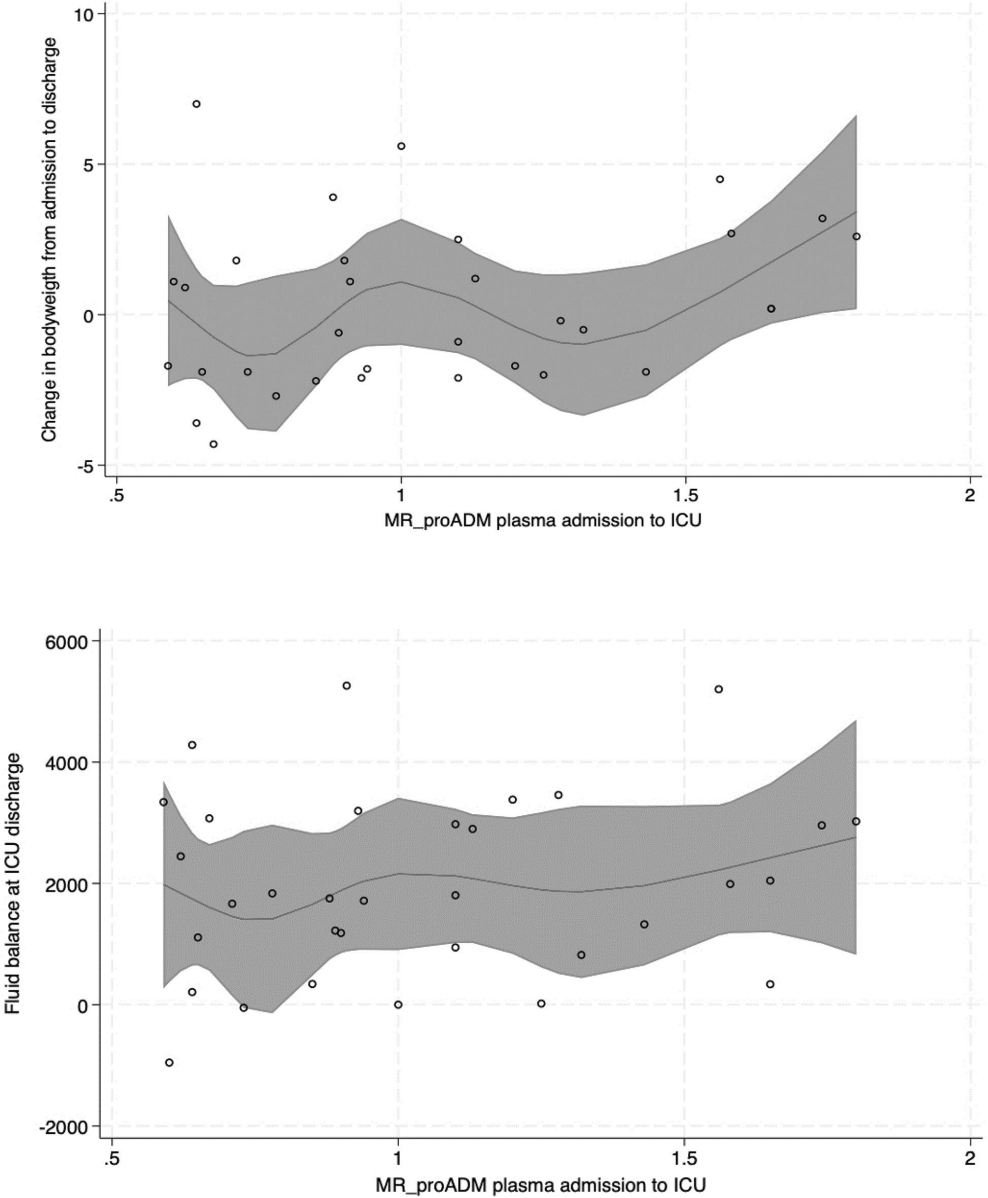
Combination of scatterplot and restricted cubic spline regression analysis of the relationship between MR-proADM at admission and fluid balance at ICU discharge. The restricted cubic spline is shown with a solid line, the grey area represents the 95% CI of the spline. Each circle represents an observation of MR-proADM.

The sensitivity analysis of MR-proADM at ICU admission and fluid balance at ICU discharge revealed an increase of 680.9 mL in fluid balance per nmol/L MR-proADM (95% CI − 803.5 to 2165, *p* = 0.357). A visualization of the fluid balance and MR-pro ADM relationship can be found in Fig. 2. Per nmol/L MR-proADM at ICU admission, the weight gain at day 6 after surgery was 1.8 kg (95% CI − 0.8 to 4.4, *p* = 167), respective Figure S1 is depicted in the Supplemental File.

### MR-proADM over time

**Fig. 3:**
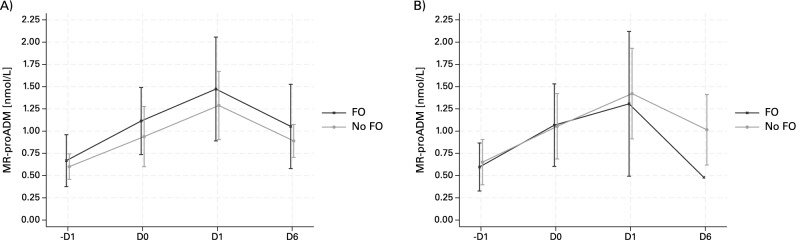
MR-proADM levels over time in patients with and without fluid overload. (**A**) Fluid overload assessed at ICU discharge. *Missing values D6: four proADM values missing (3 in the FO group, 1 in the no FO group).* (**B**) Fluid overload assessed at day 6 post surgery. *Missing values D6: four proADM values missing (2 in the FO group, 2 in the no FO group).*

Change in MR-proADM levels perioperatively was not significant different in patient with or without FO (median MR-proADM difference 0.29 nmol/L (IQR 0.22–0.74) versus 0.4 (IQR 0.22–0.69), *p* = 0.3). Median MR-proADM levels were: -D1 0.58 nmol/L (IQR 0.51–0.69), D0 0.94 nmol/L (IQR 0.73–1.28), at D1 (i.e. ICU discharge) 1.34 nmol/L (IQR 1.10–1.73), and D6 0.9 nmol/L (IQR 0.70– 1.13). Time series analysis showed a non-significant trend towards higher MR-pro ADM values in the FO group after surgery that continues until post-operative day 1 (ICU discharge) and then declines (see Fig. 3).

### Further outcomes

There was no significant association of MR-proADM level at ICU admission or duration of mechanical ventilation or duration or dose of vasopressors. Exploratory analyses revealed no significant association of MR-proADM and the duration of mechanical ventilation (duration of mechanical ventilation increased 0.14 days per nmol/L MR-proADM (95% CI − 0.04 to 0.33, *p* = 0.115). For vasopressor dose, we found an increase of 1 mg norepinephrine per nmol/L MR-proADM (95% CI − 1.5 to 3.6, *p* = 0.4).

## Discussion

Our exploratory post-hoc analysis of the HERACLES randomized controlled trial on MR-proADM in a consecutive and representative subset of patients undergoing elective cardiac surgery showed no association of MR-proADM levels at ICU admission and fluid overload at ICU discharge or on day six post-surgery. Time series analysis shows elevated levels of MR-pro ADM in the FO group after surgery with continued increase until post-operative day 1.

Fluid overload is common after cardiac surgery and is a key factor for delay in recovery and several clinically important complications in patients undergoing open heart surgery^9,32–34^. The main factors contributing to FO in this population is overt fluid administration and capillary leakage secondary to inflammatory response after extracorporeal circulation and hemodynamic alterations^32,35^. ADM levels in the tissues and plasma are elevated as a response to pathologic conditions such as hypertension, tissue hypoxia or hypervolemia, and cytokines (e.g. tumor necrosis factor (TNF)-α, and interleukin (IL)-1) trigger release of ADM in vascular endothelial and smooth muscle cells^19,36^. Therefore, ADM could play an important role in risk prediction models for morbidity and mortality in patients undergoing cardiac surgery^37–40^. It is therefore not surprising that there are efforts to combine novel biomarkers with existing risk scores, such as the EuroSCORE^41^. One study observed, that MR-proADM in combination with EuroSCORE significantly improves the prediction of 30-day compared to EuroSCORE alone^42^.

For clinical practice, it seems even more important to have valid predictors for patient-centred outcomes, such as prolonged mechanical ventilation, need for renal replacement therapy, and/or fluid overload to develop patient-tailored treatment on the ICU. However, with regard to fluid overload in this patient population, our study showed a poor predictive value of MR-proADM. This could be solely explained due to a limited sample size of our study, and thus lack of power to test the hypothesis (under-powering). However, Paasen et al.^43^ could show in a rather similar sized population of forty cardiac-surgery patients, that perioperative MR-proADM was associated with development of acute respiratory distress syndrome (ARDS) after cardiac-surgery (cut-off > 1.5 nmol/L).

Another reason for the negative result of our study could be selection of our patient population (selection bias), which is a relatively low-risk patient population with preserved or only slightly reduced ejection fraction, without severe renal failure and rather short ICU stay. And although two-thirds of our patient population fulfilled the definition of FO at ICU discharge, the clinical impact might be somewhat negligible in this relatively health population. A similar patient population was included in a study by Holms et al.^21^ who investigated the dynamics of MR-proADM during cardiac surgery. Comparing the time course of MR-proADM values in our study with those in the study by Holmes et al., it appears that our study population follows normal dynamics of cardiac surgery patients with a rather uneventful postoperative course. Although patients who develop FO on discharge from the ICU tend to have higher scores, but we were unable to demonstrate statistical significance. Thus, changes in MR-proADM values may correlate with CBP-associated effects rather than the occurrence of fluid overload.

This might put the reliability of the biomarker itself in question, i.e. whether a marker that is activated both by surgery itself and by exposure to the CPB circuit can reliably be used to predict a phenomenon attributable to endothelial injury. Nevertheless, although studies show significant different levels of MR-proADM in patients with vasoplegic shock after cardiac surgery compared to patients with uncomplicated coronary artery bypass graft^40^, the potential prolonged vasodilatation early is unknown.

The fact that higher levels of MR-proADM were observed in patients that are deemed fluid overloaded at ICU discharge might indicate that patients with increased inflammatory biotrauma following surgery and/or cardiopulmonary bypass might be at risk to develop FO post cardiac surgery.

## Limitations

This trial has several important limitations that warrant discussion. The results presented here are of exploratory character and lack statistical power due to limited sample size. Thus, our results may only hint towards the presence, respectively absence of effects. Additionally, due to the rather limited sample size it was not sensible to adjust the logistic regression analysis for important confounders. Further, all limitations applied to the HERACLES randomized controlled trial including its single centre design and the exclusion of patients with relevant organ dysfunctions from the trial apply to the present study^3,27^. Further, FO at ICU discharge was calculated from total balance per body weight, and at day 6 from body weight measured. In addition, there was no formally standardised assessment of post-ICU fluid intake and output, as well as administration of diuretics on the ward (after ICU discharge).

## Conclusions

Our results reveal that MR-proADM at ICU admission is not associated with fluid overload at ICU discharge in patients post elective cardiac surgery. The trajectory of MR-proADM levels did not significantly differ between the two study groups (i.e. fluid overload vs no fluid overload), but patients with FO had elevated MR-proADM levels.

## Supplementary Information

Supplementary Information.

## Data Availability

The data is available upon reasonable non-commercial request from the corresponding author, Anna S. Messmer.
