# Supplementary material for: The association of midregional pro-adrenomedullin (MR-proADM) at ICU admission and fluid overload in patients post elective cardiac surgery
Source: Sci Rep. 2024 Sep 8;14:20897. doi: 10.1038/s41598-024-71918-x (PMC11381535; doi:10.1038/s41598-024-71918-x)
Supplement: Supplementary file 1 — Supplementary Information. [file 41598_2024_71918_MOESM1_ESM.docx]

Supplemental Digital Content

This appendix has been provided by the authors to provide additional information

Supplement to: Pfortmueller CA, Ott I, Müller M, Wilson D, Schefold JC, Messmer AS

**The association of midregional pro-adrenomedullin at intensive care unit admission and fluid overload in patients post elective cardiac surgery**

*Corresponding author

Anna Messmer, MD

Department of Intensive Care,

Inselspital, Bern University Hospital and University of Bern

Freiburgstrasse 10

3010 Bern

Switzerland

ORCID: 0000-0002-3206-9112

**The association of midregional pro-adrenomedullin at intensive care unit admission and fluid overload in patients post elective cardiac surgery**

**Table of Contents**

[Table S1. Comparison of General Studypopulation and MR-proADM Subpopulation. 3](#_Toc173755648)

[Table S2. Logistic regression analysis of MR-proADM at ICU admission and fluid overload at ICU discharge 4](#_Toc173755649)

[Table S3. Logistic regression analysis of MR-proADM at ICU admission and fluid overload at ICU discharge, adjusted for the randomisation group 4](#_Toc173755650)

[Table S4. Logistic regression analysis of MR-proADM at ICU admission and fluid overload at day 6 postsurgery 5](#_Toc173755651)

[Table S5. Logistic regression analysis of MR-proADM at ICU admission and fluid overload at day 6 postsurgery, adjusted for the randomisation group 5](#_Toc173755652)

[Table S6. Linear regression analysis of MR-proADM at ICU admission and fluid balance at ICU discharge 6](#_Toc173755653)

[Table S7. Linear regression analysis of MR-proADM at ICU admission and weight gain at day 6 post surgery 6](#_Toc173755654)

[Table S8. Mixed-effect restricted maximal likelihood (RML) regression of proADM levels over time in patients with and without fluid overload at ICU discharge 7](#_Toc173755655)

[Table S9. Linear regression analysis of MR-proADM at ICU admission and duration of mechanical ventilation (in days) 7](#_Toc173755656)

[Table S10. Linear regression analysis of MR-proADM at ICU admission and dose of norephinephrine (in mg) 8](#_Toc173755657)

[Figure S1. Combination of scatterplot and restricted cubic spline regression analysis of the relationship between MR-proADM at admission and weight gain at day 6 post surgery 8](#_Toc173755658)

## Table S1. Comparison of General Studypopulation and MR-proADM Subpopulation.

| **Characteristics** | **General Studypopulation**  **(**N = 165^1^) | **MR-proADM  Subpopulation**  (N = 33^1^) | **P-value** |
| --- | --- | --- | --- |
| **Demographics** |  |  |  |
| Sex  male  female | 131 (79.4%)  34 (20.6%) | 26 (78.8%)  7 (21.2%) | 0.937 |
| Age (years) | 66.0 (61.0, 72.0) | 67.0 (59.0, 72.0) | 0.767 |
| Height | 173 (168.0, 178.0) | 175 (170, 178) | 0.660 |
| Weight, baseline | 81 (72.0, 91.8) | 80 (73.0, 95.0) | 0.747 |
| BMI, baseline | 26.4 (24.5, 30.8) | 27.0 (24.7, 30.9) | 0.711 |
| Randomisation group |  |  | 0.269 |
| *Hypertonic Saline* | 53 (32.1%) | 14 (42.4%) |  |
| *Normal Saline* | 48 (29.1%) | 11 (33.3%) |  |
| *Standard of Care* | 64 (38.8%) | 8 (24.2%) |  |
| ASA classification |  |  | 0.654 |
| *3* | 1 (0.6%) | 0 (0%) |  |
| *4* | 164 (99.4%) | 33 (100.0%) |  |
| **Comorbidities** |  |  |  |
| LVEF (%) at baseline | 60 (55; 65) | 60 (50; 65) | 0.753 |
| Known chronic renal disease | 22 (13.3%) | 2 (6.1%) | 0.243 |
| **Surgery/Anaesthesia Characteristics** |  |  |  |
| Duration anaesthesia (h) | 5.4 (4.5, 6.2) | 5.4 (4.9, 6.1) | 0.547 |
| Duration surgery (h) | 3.9 (3.1, 4.6) | 3.6 (3.1, 4.5) | 0.573 |
| Duration ECC | 1.38 (1.17, 2.09) | 1.40 (1.28, 2.37) | 0.200 |
| **Post-surgery characteristics** |  |  |  |
| Percentage of FO (day 1) | 5.5 (3.2, 7.6) | 6.1 (4.9, 8.1) | 0.140 |
| FO at day 1 | 91 (55.5%) | 22 (66.7%) | 0.236 |
| FB at day 1 | 1096 (209, 2.439) | 1.752 (944, 3.022) | 0.052 |
| Cumulative Fluids perioperative | 7918 (5.967, 9.334) | 8.078 (5776, 9.317) | 0.824 |
| Duration of MV (d) | 0.6 (0.4, 0.7) | 0.6 (0.5, 0.6) | 0.855 |
| Duration of vasopressors (h) | 7.4 (5.5, 17.8) | 6.9 (5.4, 17.3) | 0.669 |
| Vasopressordose (mg) | 0.35 (0.10, 2.06) | 0.31 (0.09, 2.04) | 0.607 |
| **Ouctomes** |  |  |  |
| LOS ICU | 0.89 (0.84, 0.92) | 0.88 (0.84, 0.91) | 0.367 |
| LOS Hospital | 8.52 (7.10, 11.00) | 8.04 (7.38, 10.98) | 0.993 |

^1^*Mean (SD); n (%); Median (IQR),*

BMI = Body Mass Index; ASA = American Society of Anaesthesiologists; LVEF = Left Ventricular Ejection Fraction; DHCA = Deep Hypothermic Circulatory Arrest; ECC = Extra Corporeal Circulation; FO = Fluid Overload; MV = Mechanical Ventilation; FB = Fluid Balance; ICU = Intensive Care Unit; LOS = Length of Stay;

## Table S2. Logistic regression analysis of MR-proADM at ICU admission and fluid overload at ICU discharge

| Logistic regression Number of obs = 33 |
| --- |
| LR chi2(1) = 1.81 |
| Prob > chi2 = 0.1784 |
| Log likelihood = -20.0997 Pseudo R2 = 0.0431 |
| ------------------------------------------------------------------------------ |
| FO_dcICU \| Odds ratio Std. err. z P>\|z\| [95% conf. interval] |
| -------------+---------------------------------------------------------------- |
| proADM_Vp2 \| 4.3 5.0 1.28 0.201 0.5 40.9 |
| _cons \| 0.4 0.5 -0.67 0.501 0.0 4.6 |
| ------------------------------------------------------------------------------ |

Note: _cons estimates baseline odds.

## Table S3. Logistic regression analysis of MR-proADM at ICU admission and fluid overload at ICU discharge, adjusted for the randomisation group

| Logistic regression Number of obs = 33 | | | | | | | |  |  |
| --- | --- | --- | --- | --- | --- | --- | --- | --- | --- |
| LR chi2(3) = 7.51 | | | | | | | |  |  |
| Prob > chi2 = 0.0572 | | | | | | | |  |  |
| Log likelihood = -17.248103 Pseudo R2 = 0.1789 | | | | | | | |  |  |
| ------------------------------------------------------------------------------ | | | | | | | |  |  |
| FO_dcICU \| Odds ratio Std. err. z P>\|z\| [95% conf. interval] | | | | | | | |  |  |
| -------------+---------------------------------------------------------------- | | | | | | | |  |  |
| proADM_Vp2 \| 9.7 12.9 1.72 0.086 0.7 130.9 | | | | | | | |  |  |
| \| |  |  |  |  |  |  |  | |  |
| group \| |  |  |  |  |  |  |  | |  |
| B \| 0.5 0.5 -0.65 0.516 0.1 3.7 | | | | | | | |  |  |
| None \| 0.1 0.1 -2.09 0.037 0.0 0.9 | | | | | | | |  |  |
| \| |  |  |  |  |  |  |  | |  |
| _cons \| 0.5 0.6 -0.58 0.563 0.0 6.0 | | | | | | | |  |  |
| ------------------------------------------------------------------------------ | | | | | | | |  |  |

Note: _cons estimates baseline odds.

## Table S4. Logistic regression analysis of MR-proADM at ICU admission and fluid overload at day 6 postsurgery

| Logistic regression Number of obs = 33 |
| --- |
| LR chi2(1) = 0.00 |
| Prob > chi2 = 0.9541 |
| Log likelihood = -10.051336 Pseudo R2 = 0.0002 |
| ------------------------------------------------------------------------------ |
| FO_dchosp \| Odds ratio Std. err. z P>\|z\| [95% conf. interval] |
| -------------+---------------------------------------------------------------- |
| proADM_Vp2 \| 1.10 1.82 0.06 0.954 0.04 28.32 |
| _cons \| 0.09 0.17 -1.29 0.198 0.00 3.51 |
| ------------------------------------------------------------------------------ |

Note: _cons estimates baseline odds.

## Table S5. Logistic regression analysis of MR-proADM at ICU admission and fluid overload at day 6 postsurgery, adjusted for the randomisation group

| Logistic regression Number of obs = 25 |  |  |  |  |  |  |  |  |  |
| --- | --- | --- | --- | --- | --- | --- | --- | --- | --- |
| LR chi2(2) = 0.73 |  |  |  |  |  |  |  |  |  |
| Prob > chi2 = 0.6952 |  |  |  |  |  |  |  |  |  |
| Log likelihood = -8.8095863 Pseudo R2 = 0.0396 |  |  |  |  |  |  |  |  |  |
| ------------------------------------------------------------------------------ |  |  |  |  |  |  |  |  |  |
| FO_dchosp \| Odds ratio Std. err. z P>\|z\| [95% conf. interval] |  |  |  |  |  |  |  |  |  |
| -------------+---------------------------------------------------------------- |  |  |  |  |  |  |  |  |  |
| proADM_Vp2 \| 1.31 2.75 0.13 0.896 0.02 79.37 |  |  |  |  |  |  |  |  |  |
| \| | |  |  |  |  |  |  |  |  |
| group \| | |  |  |  |  |  |  |  |  |
| B \| 2.81 3.69 0.78 0.433 0.21 37.00 |  |  |  |  |  |  |  |  |  |
| None \| 1.00 (empty) | | | | |  |  |  |  |  |
| \| | |  |  |  |  |  |  |  |  |
| _cons \| 0.06 0.14 -1.21 0.224 0.00 5.68 |  |  |  |  |  |  |  |  |  |
| ------------------------------------------------------------------------------ |  |  |  |  |  |  |  |  |  |

Note: _cons estimates baseline odds.

## Table S6. Linear regression analysis of MR-proADM at ICU admission and fluid balance at ICU discharge

| Source \| SS df MS Number of obs = 33 |
| --- |
| -------------+---------------------------------- F(1, 31) = 0.88 |
| Model \| 2012562.99 1 2012562.99 Prob > F = 0.3568 |
| Residual \| 71291139.6 31 2299714.18 R-squared = 0.0275 |
| -------------+---------------------------------- Adj R-squared = -0.0039 |
| Total \| 73303702.6 32 2290740.71 Root MSE = 1516.5 |
| ------------------------------------------------------------------------------ |
| fluid_bala~c \| Coefficient Std. err. t P>\|t\| [95% conf. interval] |
| -------------+---------------------------------------------------------------- |
| proADM_Vp2 \| 680.9 727.8 0.94 0.357 -803.5 2165.2 |
| _cons \| 1245.6 812.1 1.53 0.135 -410.6 2901.8 |
| ------------------------------------------------------------------------------ |

## Table S7. Linear regression analysis of MR-proADM at ICU admission and weight gain at day 6 post surgery

| Source \| SS df MS Number of obs = 33 |
| --- |
| -------------+---------------------------------- F(1, 31) = 2.00 |
| Model \| 14.1534476 1 14.1534476 Prob > F = 0.1670 |
| Residual \| 219.108977 31 7.06803151 R-squared = 0.0607 |
| -------------+---------------------------------- Adj R-squared = 0.0304 |
| Total \| 233.262424 32 7.28945076 Root MSE = 2.6586 |
| ------------------------------------------------------------------------------ |
| ch_bw_hosp \| Coefficient Std. err. t P>\|t\| [95% conf. interval] |
| -------------+---------------------------------------------------------------- |
| proADM_Vp2 \| 1.8 1.3 1.42 0.167 -0.8 4.4 |
| _cons \| -1.7 1.4 -1.16 0.253 -4.6 1.2 |
| ------------------------------------------------------------------------------ |

## Table S8. Mixed-effect restricted maximal likelihood (RML) regression of proADM levels over time in patients with and without fluid overload at ICU discharge

| Mixed-effects REML regression Number of obs = 128 |  |  |  |  |  |  |  |  |  |
| --- | --- | --- | --- | --- | --- | --- | --- | --- | --- |
| Group variable: study_id Number of groups = 33 |  |  |  |  |  |  |  |  |  |
| Obs per group: | | | | | | | |  |  |
| min = 3 |  |  |  |  |  |  |  |  |  |
| avg = 3.9 |  |  |  |  |  |  |  |  |  |
| max = 4 |  |  |  |  |  |  |  |  |  |
| Wald chi2(7) = 154.98 |  |  |  |  |  |  |  |  |  |
| Log restricted-likelihood = -45.459163 Prob > chi2 = 0.0000 |  |  |  |  |  |  |  |  |  |
| ----------------------------------------------------------------------------------------- | |  |  |  |  |  |  |  |  |
| proADM_Vp \| Coefficient Std. err. z P>\|z\| [95% conf. interval] | |  |  |  |  |  |  |  |  |
| ------------------------+---------------------------------------------------------------- | |  |  |  |  |  |  |  |  |
| FO_dcICU \| | | |  |  |  |  |  |  |  |
| No \| 0 (base) | | | | |  |  |  |  |  |
| Yes \| .0672727 .146933 0.46 0.647 -.2207107 .3552561 | |  |  |  |  |  |  |  |  |
| \| | | |  |  |  |  |  |  |  |
| measure_proADM \| | | |  |  |  |  |  |  |  |
| -D1 \| 0 (base) | | | | |  |  |  |  |  |
| D0 \| .3372727 .1076096 3.13 0.002 .1263619 .5481836 | |  |  |  |  |  |  |  |  |
| D1 \| .6872727 .1076096 6.39 0.000 .4763619 .8981836 | |  |  |  |  |  |  |  |  |
| D6 \| .2935213 .1109795 2.64 0.008 .0760055 .511037 | |  |  |  |  |  |  |  |  |
| \| | | |  |  |  |  |  |  |  |
| FO_dcICU#measure_proADM \| | | |  |  |  |  |  |  |  |
| Yes#D0 \| .1081818 .1317943 0.82 0.412 -.1501302 .3664938 | |  |  |  |  |  |  |  |  |
| Yes#D1 \| .1172727 .1317943 0.89 0.374 -.1410393 .3755847 | |  |  |  |  |  |  |  |  |
| Yes#D6 \| .0875338 .1367037 0.64 0.522 -.1804005 .3554681 | |  |  |  |  |  |  |  |  |
| \| | | |  |  |  |  |  |  |  |
| _cons \| .6009091 .1199703 5.01 0.000 .3657716 .8360466 | |  |  |  |  |  |  |  |  |
| ----------------------------------------------------------------------------------------- | |  |  |  |  |  |  |  |  |

## Table S9. Linear regression analysis of MR-proADM at ICU admission and duration of mechanical ventilation (in days)

| Source \| SS df MS Number of obs = 33 |
| --- |
| -------------+---------------------------------- F(1, 31) = 2.63 |
| Model \| .095970969 1 .095970969 Prob > F = 0.1152 |
| Residual \| 1.132326 31 .036526645 R-squared = 0.0781 |
| -------------+---------------------------------- Adj R-squared = 0.0484 |
| Total \| 1.22829697 32 .03838428 Root MSE = .19112 |
| ------------------------------------------------------------------------------ |
| duration_M~d \| Coefficient Std. err. t P>\|t\| [95% conf. interval] |
| -------------+---------------------------------------------------------------- |
| proADM_Vp2 \| 0.15 0.09 1.62 0.115 -0.04 0.34 |
| _cons \| 0.42 0.10 4.07 0.000 0.21 0.62 |
| ------------------------------------------------------------------------------ |

## Table S10. Linear regression analysis of MR-proADM at ICU admission and dose of norephinephrine (in mg)

| Source \| SS df MS Number of obs = 22 |
| --- |
| -------------+---------------------------------- F(1, 20) = 0.74 |
| Model \| 2.81409232 1 2.81409232 Prob > F = 0.3998 |
| Residual \| 76.0478692 20 3.80239346 R-squared = 0.0357 |
| -------------+---------------------------------- Adj R-squared = -0.0125 |
| Total \| 78.8619615 21 3.7553315 Root MSE = 1.95 |
| ------------------------------------------------------------------------------ |
| dose_vasomg \| Coefficient Std. err. t P>\|t\| [95% conf. interval] |
| -------------+---------------------------------------------------------------- |
| proADM_Vp2 \| 1.06 1.24 0.86 0.400 -1.51 3.64 |
| _cons \| 0.10 1.36 0.07 0.943 -2.75 2.95 |
| ------------------------------------------------------------------------------ |

## Figure S1. Combination of scatterplot and restricted cubic spline regression analysis of the relationship between MR-proADM at admission and weight gain at day 6 post surgery





The restricted cubic spline is shown with a solid line, the grey area represents the 95% CI. Each circle represents an observation.
